# Environmental differences explain subtle yet detectable genetic structure in a widespread pollinator

**DOI:** 10.1186/s12862-022-01963-5

**Published:** 2022-02-01

**Authors:** Marcel Glück, Julia C. Geue, Henri A. Thomassen

**Affiliations:** 1 Comparative Zoology, Institute of Evolution and Ecology, Tübingen University, Tübingen, Germany; 2 Department of Ecology and Environmental Sciences, Umeå University, Umeå, Sweden

**Keywords:** Landscape genetics, Population genetics, Quasi-panmixia, Environmental gradients, Isolation by environment, Microsatellites, Generalized dissimilarity modelling, Eastern Europe, Bumblebee, *Bombus terrestris*

## Abstract

**Background:**

The environment is a strong driver of genetic structure in many natural populations, yet often neglected in population genetic studies. This may be a particular problem in vagile species, where subtle structure cannot be explained by limitations to dispersal. Consequently, these species might falsely be considered quasi-panmictic and hence potentially mismanaged. A species this might apply to, is the buff-tailed bumble bee (*Bombus terrestris*), an economically important and widespread pollinator, which is considered to be quasi-panmictic at mainland continental scales. Here we aimed to (i) quantify genetic structure in 21+ populations of the buff-tailed bumble bee, sampled throughout two Eastern European countries, and (ii) analyse the degree to which structure is explained by environmental differences, habitat permeability and geographic distance. Using 12 microsatellite loci, we characterised populations of this species with Fst analyses, complemented by discriminant analysis of principal components and Bayesian clustering approaches. We then applied generalized dissimilarity modelling to simultaneously assess the informativeness of geographic distance, habitat permeability and environmental differences among populations in explaining divergence.

**Results:**

Genetic structure of the buff-tailed bumble bee quantified by means of Fst was subtle and not detected by Bayesian clustering. Discriminant analysis of principal components suggested insignificant but still noticeable structure that slightly exceeded estimates obtained through Fst analyses. As expected, geographic distance and habitat permeability were not informative in explaining the spatial pattern of genetic divergence. Yet, environmental variables related to temperature, vegetation and topography were highly informative, explaining between 33 and 39% of the genetic variation observed.

**Conclusions:**

In contrast to previous studies reporting quasi-panmixia in continental populations of this species, we demonstrated the presence of subtle population structure related to environmental heterogeneity. Environmental data proved to be highly useful in unravelling the drivers of genetic structure in this vagile and opportunistic species. We highlight the potential of including these data to obtain a better understanding of population structure and the processes driving it in species considered to be quasi-panmictic.

**Supplementary Information:**

The online version contains supplementary material available at 10.1186/s12862-022-01963-5.

## Background

Detecting genetic population structure and its underlying causes is crucial to better understand basic evolutionary ecological processes and how these are affected by human actions, as well as to improve conservation strategies. Population structure is often inferred using methods that are only based on genetic data, and do not take into account the geographic relationships between populations [1]. These methods perform well when population structure is strong, but may fail to correctly detect weak structure. Considering spatial relationships can help to improve the detectability of weak structure [2–4], but mainly when there are small but distinct genetic breaks in geographic space [5]. Gradually changing genetic population structure is, however, notoriously difficult to detect, which might even be further complicated when structure is subtle (e.g. [5, 6]). As a consequence, species exhibiting such patterns might incorrectly be considered quasi-panmictic.

Population structure results of a balance between gene flow, genetic drift, natural selection, and mutation. When dispersal becomes difficult, gene flow is reduced. This may be the case with increasing distance, or when habitats become difficult to traverse, resulting in patterns of genetic divergence known as isolation by distance (IBD, [7]) and isolation by resistance (IBR, [8]). Moreover, gene flow may also be reduced due to decreased fitness of dispersing individuals that are maladapted to new environmental conditions they encounter [9]. Following a decrease in genetic connectivity, populations may start to diverge by means of genetic drift, eventually resulting in selective processes becoming apparent in per se neutral markers as a pattern of isolation by environment/ecology (IBE, [10, 11]) or isolation by adaptation (IBA, [12]). Approaches that neglect environmental heterogeneity as a driver of population structure may thus be overly simplistic and result in an incomplete picture of the processes that structure natural populations (e.g. [13, 14]).

To this end, the field of landscape genetics [15–17] provides the tools and data to not only analyse genetic information in a spatially explicit context, but to also consider local environmental conditions and those of the habitat matrix between populations in explaining non-random gene flow across the landscape [9]. Thus, it has become possible to study the full range of evolutionary ecological processes driving population divergence and to tease apart their relative importance.

Integrating environmental dissimilarities into the analysis of population structure is particularly promising in species capable of dispersing widely. Typically, these species do not show genetic patterns consistent with IBD or IBR, and exhibit divergence levels close to panmixia [18, 19], which we define as random mating [20] and, consequently, the absence of genetic subdivision. Such a species, which is deemed quasi-panmictic at the subspecies level, is the buff-tailed bumble bee (*Bombus terrestris*). This highly polymorphic pollinator occurs across various environmental gradients [21, 22], and morphological differences have prompted a division in nine subspecies [23]. Throughout the European continent [21, 24] and Tasmania [22], the species exhibits a remarkable niche breadth and is present across a wide range of habitats that differ strikingly in precipitation patterns, altitude and vegetation. Yet, studies on mainland populations have inferred little to no genetic divergence at various spatial scales, irrespective of the genetic marker applied. Using polymorphic microsatellites, no significant structure was observed at either fine [25] or broad scales [26]. A comparable picture emerged for broad-scale studies using mitochondrial (mt) DNA [27, 28], and single nucleotide polymorphisms [27]. These results are unlikely to be artefacts of the molecular markers used, as populations separated by strong oceanic barriers between islands or continents exhibited significant divergence in microsatellites [26, 28, 29], mtDNA [29] and phenotypic traits [30, 31]. Quasi-panmixia in this species may be the result of its high vagility. For instance, after its introduction to Tasmania, it only took seven years for the species to spread across the entire island [32] and buff-tailed bumble bee queens from introduced populations in Chile have been shown to disperse up to 200 km per year from their hibernation site [33]. Although gene flow in this haplodiploid species, where workers are not reproducing and males are haploid, is largely a function of queen dispersal [34], males have also been reported to disperse up to 10 km [35]. Only large water bodies and strong winds have so far been implied to limit gene flow [28]. Interestingly, however, the potential for subtle population structure and how it might be influenced by environmental heterogeneity remain unelucidated.

This might pose a problem in the face of climate change, where species might show pronounced distribution shifts (e.g. [24, 36]). Currently, the future distribution of species is predicted using known associations between today’s species presence and environmental variables. Undoubtedly, this approach holds value as it might allow the delineation of areas that protect both current and future suitable habitats (e.g. [37, 38]). Yet, these models are uninformative about landscape-induced changes in genetic composition that might accompany distribution shifts. Hence, species persistence is only modelled correctly if the influence of the environment on the genetic composition of populations is known. This maxim might also apply to widespread and quasi-panmictic species such as the buff-tailed bumble bee, where climate change-induced range losses might isolate populations genetically that are currently well connected [24].

Here we aimed to quantify population structure and its drivers in the buff-tailed bumble bee across two countries that exhibit pronounced landscape heterogeneity that is readily exploited by this species [21, 24]. Indeed, as pronounced environmental heterogeneity might increase the likelihood of individuals to experience post-dispersal environmental conditions they are maladapted to, we hypothesised that this species shows genetic structuring consistent with a scenario of IBE [14, 39]. Further, as the buff-tailed bumble bee is highly vagile [22, 35]*,* we expected geographic distance and landscape resistance to play only minor roles in explaining population divergence.

We used both spatially implicit and explicit analyses to identify the most likely drivers of population structure. Deploying 12 highly polymorphic microsatellite loci, we did not detect population differentiation using spatially implicit Bayesian clustering. However, Fst analyses demonstrated subtle yet marginally significant genetic divergence among populations, a finding that was partially corroborated using discriminant analysis of principal components (DAPC, [40]). We further used generalized dissimilarity modelling (GDM, [41]) to simultaneously assess how geographic distance, landscape resistance, and environmental dissimilarities among populations shape genetic divergence. Most notably, only environmental dissimilarities proved to be informative in detecting and unravelling the drivers of genetic structure in this widespread and abundant pollinator.

## Results

### Genotyping and exclusions

A total of 376 out of 385 buff-tailed bumble bees were successfully genotyped at 12 microsatellite loci (Additional file 1: Table S1), with locus-specific error rates ranging between 0 and 5.56%. Using GenAlEx [42, 43] and Colony [44], we detected clones and full siblings that might interfere with obtaining accurate estimates of genetic population structure. In total, we excluded 8 clones, 19 full siblings, and 2 individuals inferred as being clones and full siblings at the same time. To prepare the data set for subsequent tests for central population-genetic assumptions, we identified putative males based on multilocus heterozygosity scores. In the end, we excluded 36 putative males of which 34 were homozygous across all 12 loci and 2 across 10 or 11 loci.

### Descriptive statistics

Using Micro-Checker [45], we detected null alleles at all except for two loci (ms66 and ms86; Additional file 1: Table S1) with numbers ranging from one to ten. Signals of stuttering were present at five loci (ms39, 80, 81, 85, and 41) in one to eight sampling sites (Additional file 1: Table S1). We did not detect signals of large allele dropout. Deviations from Hardy–Weinberg equilibrium (HWE) were observed using Genepop on the Web [46], with 8 out of 12 loci showing significant departure from HWE in one to five populations. We inferred overall significant (*P* = 0.027) linkage disequilibrium (LD) for the locus pair ms39–ms80, which was, however, not supported by population pairwise analyses, where significant LD was detected only in the populations Drӑgusani and Valea Hotarului. As null alleles, stuttering, and deviations from both HWE and LD were not consistent across either loci or populations, we retained all loci in the data set.

Observed heterozygosity (H_O_) ranged from 0.46 to 0.60 in a data set containing diploid females only (diploid data set; ‘dpds’) (Additional file 1: Table S2). After correcting for unequal sample sizes by rarefaction for seven individuals, the number of alleles ranged from 2.70 to 3.09 for ‘dpds’ and from 2.26 to 3.08 for a data set comprising haploid males and diploid females (mixed-ploidy data set; ‘mpds’) (Additional file 1: Table S2). Overall population divergence measured as Fst was low in both data sets; 0.006 (*P* = 0.02) and 0.041 (upper 95% CI: 0.047) for ‘dpds’ and ‘mpds’, respectively. Fst values among pairs of sampling sites ranged from 0 to 0.07 (‘dpds’, Additional file 1: Table S3) and from 0.01 to 0.12 (‘mpds’, Additional file 1: Table S4). Following false discovery rate (FDR, [47]) correction, five pairwise comparisons remained marginally significant (*P* = 0.053–0.059) in ‘dpds’ with Fst values between 0.046 and 0.065. None of the ‘mpds’ Fst values surpassed its corresponding upper 95% confidence interval. Pairwise and global Fst estimates in ‘dpds’ calculated with polysat [48] were higher (global = 0.039, pairwise: 0.01–0.11; Additional file 1: Table S5) than corresponding estimates computed in GenAlEx. However, neither global nor pairwise estimates exceeded their corresponding 95% confidence intervals, indicating still non-significant population differentiation. This observed discrepancy between Fst estimates calculated with GenAlEx and polysat seems to be an artefact of the different computational approaches. polysat calculates Wright’s Fst [49], based on allele frequencies derived with the ‘simpleFreq’ function that might underestimate common and overestimate rare allele frequencies (polysat manual, [48]). GenAlEx, however, estimates Fst using an approach that among others corrects for finite sample sizes and the number of populations sampled [50].

### Genetic clustering

To complement Fst analyses in inferring population divergence, we used the Bayesian clustering algorithm implemented in the program Structure [1]. Structure reconstructs patterns of genetic differentiation by assigning individuals to a specified number of clusters (*K*). Visual interpretation of the results computed using the ‘Correlated Allele Frequency model’ [51] with population IDs as priors suggested the absence of clear genetic structure (*K* = 1), irrespective of whether or not admixture was assumed. This pattern was consistently inferred across all independent runs for each *K*, ranging from 2 to 25 (Additional file 2: Figs. S1, S2). Excluding populations with fewer than 10 individuals (‘pop10’) for the ‘Admixture’ runs did not change this conclusion, regardless of whether individuals were initialised from their respective populations or not (results available on Dryad).

**Fig. 1:**
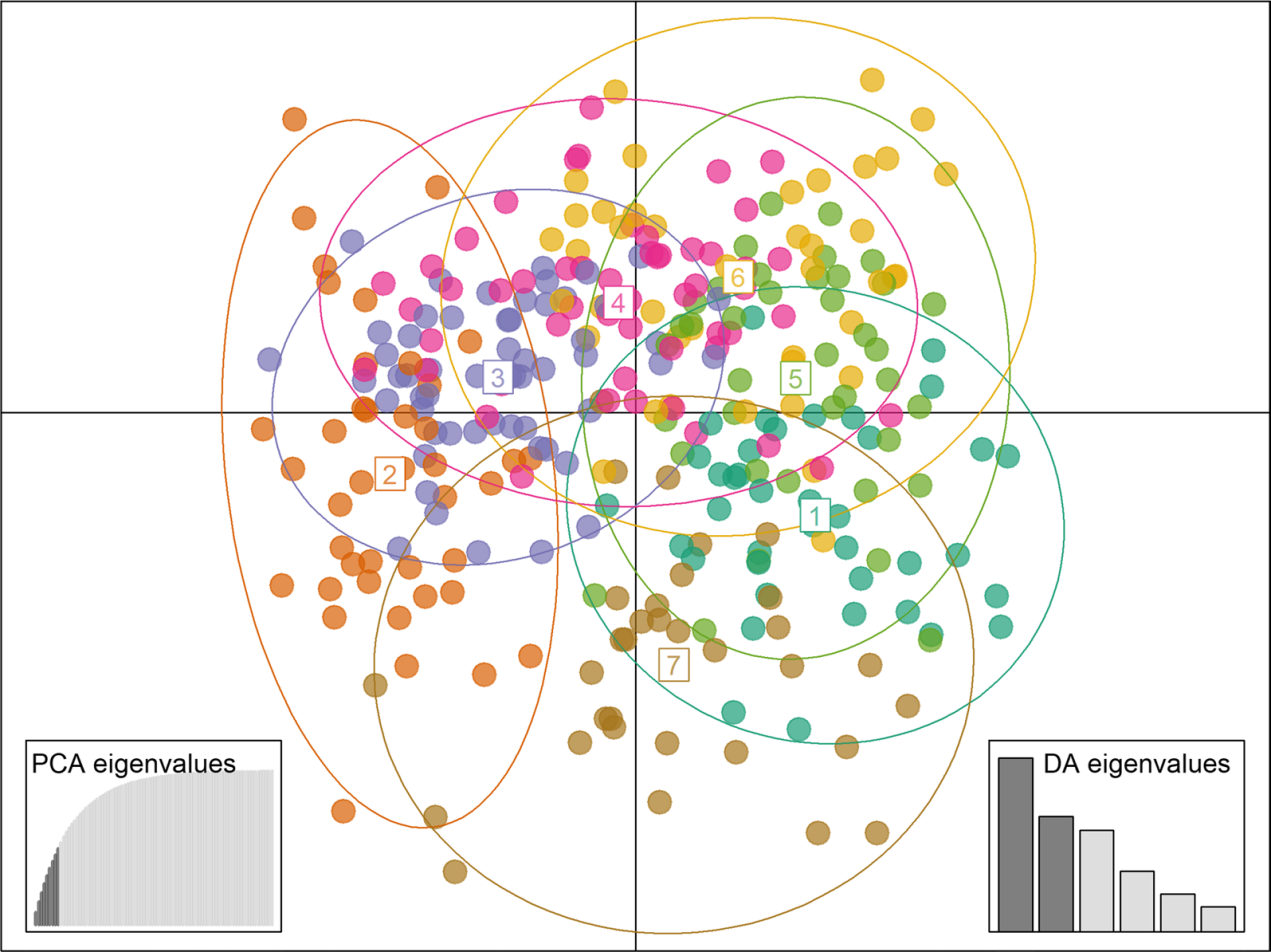
Scatter plot of the discriminant analysis of principal components using clusters identified de novo. Nine principal components (PCs) were retained to avoid overfitting, resulting in a mean a-score of 0.71. Ellipses indicate the 95% interval of assignment. Insets depict the principal component analysis (PCA) and discriminant analysis (DA) eigenvalues. Highlighted bars in insets show the number of PCs retained and the discriminant functions visualised, respectively

Because the performance of Bayesian clustering depends on how well the genetic data conform to explicit population genetic models [40] and to further assess the potential for weak genetic structure, we performed discriminant analysis of principal components (DAPC). In a run with populations as a priori groups, results obtained using DAPC were in line with the ones obtained using Structure. Following a-score optimisation to avoid overfitting, we retained 27 principal components (PCs) (Additional file 2: Fig. S3), resulting in a mean a-score of 0.04. Clusters produced overlapped significantly (Additional file 2: Fig. S4), suggesting the absence of clear genetic structuring when populations were used as prior. However, this picture changed slightly when clusters were inferred de novo; from 700 independent cluster runs performed, 648 converged successfully and deemed *K* = 7 as the most meaningful number of clusters (Additional file 2: Fig. S5). Following a-score optimisation, nine PCs were retained (Additional file 2: Figure S3), resulting in a mean a-score of 0.71. Generally, even though clusters still showed noticeable overlap (Fig. 1), structuring was more pronounced than in the analysis that had used populations as priors. Individuals within clusters originated from many populations throughout Romania and Bulgaria (Additional file 1: Table S6), suggesting admixture on a large spatial scale. Still, as DAPC with populations as prior recreate Fst-derived patterns of genetic structure [52], we concluded that clusters inferred de novo detected slight but unique genetic subdivision that had not been inferred by Fst analyses.

Despite the potential occurrence of two subspecies (*B. t. dalmatinus* and *B. t. terrestris)* native to Romania and Bulgaria [53], it seems unlikely that *B. t. terrestris* was present among our samples, as its known range is limited to the western border regions. Additionally, we would expect its presence to result in pronounced clustering, something we did not observe here. In summary, while Bayesian clustering suggested that the buff-tailed bumble bee resembles a quasi-panmictic population across Romania and Bulgaria, DAPC using clusters identified de novo suggested weak but still noticeable genetic subdivision undetected by Fst analyses.

### Landscape genetic analyses

We estimated the relative effects of potential drivers of structure using generalized dissimilarity modelling (GDM). Local habitat conditions were characterised using a set of topographical, climate and vegetation variables, and resistance distances were based on a species distribution model (SDM) generated previously [21]. GDMs explained ~ 33.5 and 39.2% of the divergence observed for ‘dpds’ and ‘mpds’, respectively (Table 1). We compared these values to those of 1000 random models. Although the random models performed surprisingly well and explained ~ 24.3 (lower CI–upper CI: 23.8–24.8) and 31.8% (31.2–32.3) of the divergence for ‘dpds’ and ‘mpds’, respectively, they were outperformed by the full models. Environmental dissimilarities contributed most to explaining differences in genetic composition. In contrast, geographic distance and the SDM-derived resistance distances performed poorly; they were not retained in the full models and only explained 0.07 and 0.00% of the total variation for ‘dpds’, and 0.00 and 0.01% for ‘mpds’ when analysed in isolation.

**Table 1:** Percentage of variance explained by the generalized dissimilarity models for the data sets encompassing diploid (‘dpds’), and both haploid and diploid (‘mpds’) individuals

	dpds	mpds
Full	33.48	39.18
Env only	33.48	39.18
Geo only	0.07	0.00
Res only	0.00	0.01
Random	24.30	31.78
Lower CI	23.77	31.24
Upper CI	24.82	32.32

Models were run with five different input data sets. (1) Full: geographic distance, resistance distance and environmental variables were included; (2) Env only, (3) Geo only, (4) Res only: contained environmental variables, geographic distance, and resistance distance only, respectively. (5) Random: mean of 1000 models with randomly generated environmental variables. Lower/Upper CI: lower/upper 95% confidence interval of the random models

**Fig. 2:**
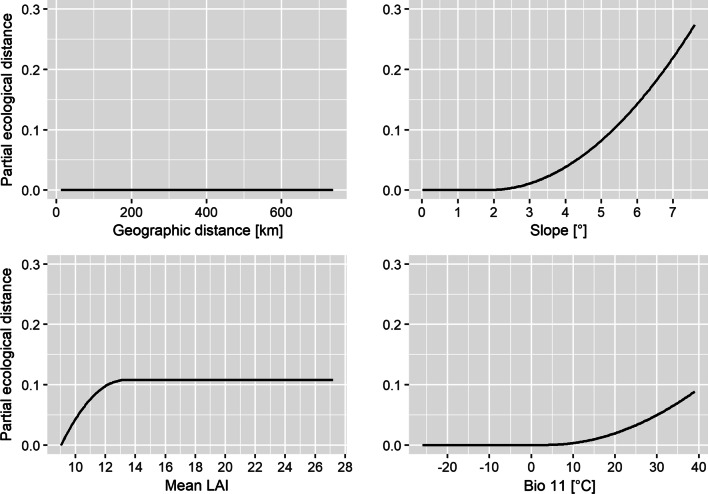
Allelic turnover across each of the environmental gradients depicted, as derived from a generalized dissimilarity model performed using environmental variables, geographic and resistance distance as predictors. Alongside geographic distance, which can be considered the baseline model of genetic structure, the splines of the three most influential variables in shaping turnover are shown; LAI: Leaf Area Index, Bio 11: mean temperature of the coldest quarter

**Fig. 3:**
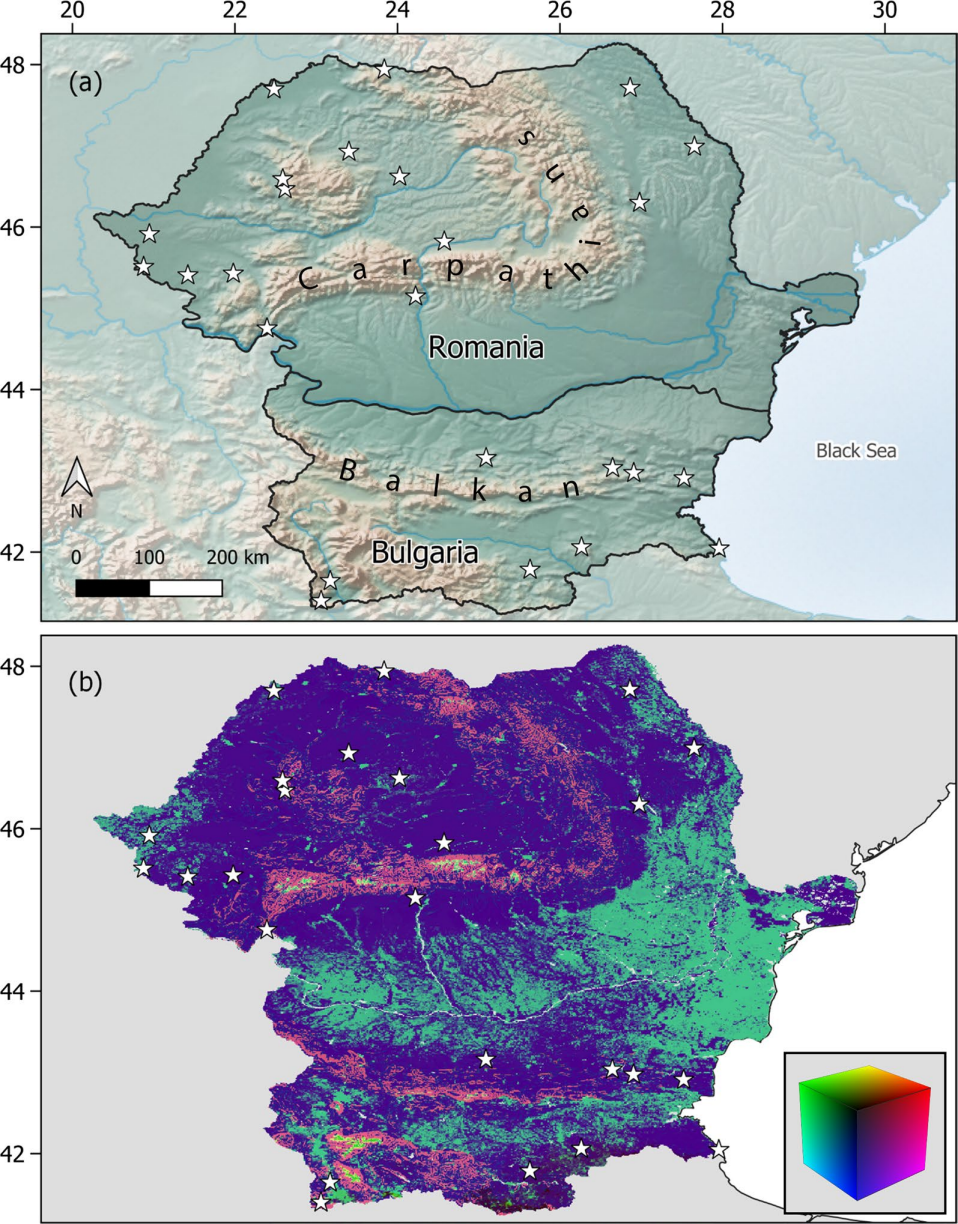
Study region and spatial generalized dissimilarity modelling prediction. **a** Location of the study area in South-Eastern Europe. Made with Natural Earth [54]. **b** Spatial patterns of the predicted genetic turnover across Romania and Bulgaria. Larger colour differences in red–green–blue colour space (see colour cube) represent higher genetic turnover. Stars mark sampling locations

The environmental variables retained included temperature, precipitation, topography, measures of surface moisture, and vegetation density. Response curves (splines) visualise how the retained environmental variables contributed to the observed genetic differences and which variables were most informative in explaining the spatial pattern of divergence (Additional file 2: Figs. S6, S7). The splines produced for predictors deemed significant were highly variable, ranging from nearly no allelic turnover across the respective environmental gradient to rapid turnover at particular gradient positions (Fig. 2; Additional file 2: Figs. S6, S7). The most important variables in both ‘dpds’ and ‘mpds’ were slope, mean Leaf Area Index (LAI) and mean temperature of the coldest quarter (Bio 11). Even among the most informative variables, pronounced differences in the splines’ shapes were apparent (Fig. 2); following an initial plateau, Bio 11 and Slope produced splines that indicated strong positive allelic turnover starting at a temperature of 9 °C and a slope of 2°, respectively. These responses differed markedly from the one shown by LAI where initially pronounced allelic turnover levelled off at a mean LAI of 13. Seasonality in surface moisture, isothermality, and mean temperature of the wettest quarter contributed as well, but their importance varied between the two data sets. Congruent to the negligible percentage of genetic differentiation explained, allelic turnover caused by geographic distance was quasi non-apparent. The map of the predicted allelic turnover across Romania and Bulgaria (Fig. 3(b)) shows that it is most pronounced along elevation gradients, such as between the Danube Delta and the Carpathians in Romania and the Balkan Mountains in Bulgaria.

## Discussion

Incorporating environmental data in population genetic studies might help to explain subtle population structure in vagile species, whose dispersal is usually not constrained by geographic distance or the habitat through which dispersal takes place. To assess the informativeness of environmental data, we quantified population structure in the widespread and abundant buff-tailed bumble bee (*Bombus terrestris*). This species is usually considered to show subtle to no structure at mainland continental scales. Using genetic data only, we inferred weak structure across Romania and Bulgaria, two countries that harbour pronounced landscape heterogeneity. Harnessing a landscape genetics approach, we related genetic divergence to this heterogeneity in the environment. Geographic distance was not informative, nor were resistance distances derived from a species distribution model that quantifies resistance to dispersal. Population structure in buff-tailed bumble bees thus follows a pattern of isolation by environment, where differences in habitat conditions reduce the fitness of dispersing individuals [9]. Thus, environmental selection against dispersers contributes to a disruption of genetic connectivity that gives rise to divergence in neutral markers through genetic drift.

The subtle level of divergence observed among Romanian and Bulgarian populations is in agreement with a previous study demonstrating weak but significant genetic structure among buff-tailed bumble bee populations in continental Europe [28]. Interestingly, some of the genetic divergence observed in the aforementioned study might simply be attributed to the inclusion of three subspecies [53]. Nonetheless, contrasting with previous work [25–27], these studies highlight the possibility of weak but significant genetic structure in this species.

More generally, low population structure has not only been detected in *B. terrestris*, but seems fairly common among *Bombus* species, including but not limited to *B. lapidarius* [55], *B. hortorum*, *B. ruderarius*, *B. soroeensis* [56] and *B. ignitus* [57]. Quasi-panmixia in these species is likely driven by extensive gene flow [22, 26, 35], strong enough to override most of the divergence caused by genetic drift and divergent natural selection. This hypothesis is in line with the absence of detectable levels of isolation by distance in our study as well as mainland populations of many other *Bombus* species, such as *B. pascuorum* [58], *B. vosnesenskii* [59], *B. lapidarius*, *B. hortorum*, *B. ruderatus *[60] and *B. flavifrons* [61]. Dispersal, and hence gene flow, may not follow a straight line, but rather a path of least resistance through suitable habitat. Resistance distances are thus often considered to be a better proxy of between-population dispersal than geographic distance. Nevertheless, our analyses suggest that gene flow is not constrained by variation in habitat permeability either, a finding potentially caused by the absence of strong oceanic barriers in the study area [62–64].

Subtle genetic structure might also result from the buff-tailed bumble bee’s generalist foraging behaviour and the presence of many workers [65–67]. The latter might increase the probability of detecting forage, while the former might increase the likelihood of it being deemed exploitable. Hence, both traits may allow this species to efficiently exploit natural and semi-natural habitats that might be devoid of related but more specialised species [68, 69]. Freed from interspecific competition, establishment success of colonies might be increased [70]. The species’ broad niche might thus translate to a more continuous distribution of nests across the landscape, with ample opportunity for gene flow among populations. However, with increasing nest densities, the benefit of a more continuous distribution might increasingly be cancelled out by strong density-induced intraspecific competition for forage [71, 72]. This competition may reduce overall nest performance and thus reproductive output [73, 74]. Hence, compared to related species, the lower nest densities observed in *B. terrestris* [60, 75, 76] might even benefit gene flow, as a relaxation of competition might allow more nests to contribute to genetic connectivity, resulting in overall weak population structure.

The subtle divergence we observed was best explained by environmental dissimilarities, in particular in the mean temperature of the coldest quarter, Leaf Area Index (LAI) and slope. The importance of temperature did not come as a surprise, as it is a predictor of the distribution of *Bombus* species [21, 77], and known to govern the emergence time of queens from hibernation [78]. Hence, queens hibernating in warmer areas might emerge earlier than those in colder areas. In fact, asynchronous emergence times may translate to phenological mismatches and thus reproductive isolation between early and late colonies. Even though laboratory experiments with *B. perplexus* and *B. lucorum* did not support this hypothesis [79, 80], the complex environment these species experience throughout their life cycles is unlikely to be fully reproduced in the laboratory [81]. Indeed, emergence patterns of sexuals in natural populations of several species, including *B. flavifrons* and *B. lucorum/terrestris*, differed strongly [82, 83], highlighting the potential for asynchronous emergence to give rise to reproductive isolation. As buff-tailed bumble bee gynes usually only mate once [84, 85], the asynchronous emergence of sexuals allows early emerging males to effectively monopolise queens, thus promoting population divergence.

Even though differences in local temperatures might thus facilitate genetic divergence, the influence of this environmental variable on the species’ life cycle is more nuanced. Facultative endothermy [86] and the ability for collective thermoregulation [87] allow nests to survive even under low temperatures [88], enabling great phenological flexibility [89]. Populations may even change from univoltinism to bivoltinism in regions hitherto considered incompatible with the latter [74]. Notably, this change in phenology seems to spread northwards into colder regions [74], a pattern coinciding with northward range shifts in this species [90]. As doubling the number of reproductive cycles per year effectively doubles the number of potential admixture events, bivoltinism might contribute to genetic homogeneity. Nevertheless, generally cold winters in Romania and Bulgaria [91, 92], and the absence of sufficient forage during the cold season might limit bivoltinism to a few inland and coastal regions in both countries [92].

Another important variable retained in the final models was mean LAI. The mechanism through which this measure of greenness is associated with genetic structure remains unclear. Yet, mean LAI was highly correlated with percent tree cover (r = 0.83, Additional file 1: Table S7), and although forests do not seem to limit bumble bee movement [93, 94], buff-tailed bumble bee queens prefer open habitat for nesting [95]. Forests may thus reduce genetic connectivity by constraining the amount of available nesting habitat [62, 96]. Moreover, assuming a negative effect of woodland on the range expansion of this species allowed to accurately model its invasion pattern in Japan [97], suggesting that queen-borne range expansion might indeed be limited by forests.

The last variable retained in our analyses was the slope of the terrain. Slope has previously been suggested to be an important determinant of suitable hibernation habitat [78, 98]. However, hibernation locations might only be of secondary importance to gene flow. Instead, the presence of suitable nesting habitat where populations are established is more likely to influence gene flow and hence genetic divergence among populations. To this end, slope might only shape genetic turnover if hibernation locations coincide with the locations where colonies are established (i.e. when queen dispersal after hibernation is strongly limited [99]). Given that buff-tailed bumble bee queens are instead highly vagile [22, 100], the mechanism of how slope governs genetic divergence remains unclear.

Although we included a large set of environmental variables, about 60% of the genetic variation remained unexplained. As we aimed to unravel the factors shaping and maintaining large-scale genetic structure, divergence explained by small-scale processes, temporal fluctuations or colony-intrinsic traits were beyond the scope of this work. Genetic divergence might also result from demographic processes, such as bottlenecks [57], which we could not cover here. Future studies might investigate the influence of the abundance and the spatial arrangement of plant species producing nectar or pollen in high quantities [81, 101, 102]. Finally, habitat alterations such as intensified farming practices might also structure populations, in particular through a synergy with natural stressors [103, 104].

## Conclusions

Seemingly panmictic populations might exhibit subtle genetic structure that can only be understood when considering the environment as a potential driver of divergence. We inferred subtle differentiation in a widespread and abundant quasi-panmictic pollinator that was not explained by geographic distance or variation in habitat permeability. Yet, using a suite of environmental variables, we showed that environmental dissimilarities are informative in explaining the observed spatial patterns of genetic structure in a highly vagile species.

## Methods

### Study species and study area

The buff-tailed bumble bee (*Bombus terrestris*) is a widespread and abundant pollinator species. Its native distribution covers much of the Palearctic realm, including Europe, North Africa, and the British and most Mediterranean and Atlantic islands [26, 29]. Its polylectic foraging and high pollination efficiency in various crops [23, 105] has rendered this species a prime candidate for pollination in the greenhouse industry where it is deployed in high numbers [106]. Upon introduction outside its native range, this species has spread rapidly and established itself in many countries including Chile, Japan and New Zealand [22, 105, 107–109], which highlights its potential to rapidly adapt to novel environments.

We conducted this study in Romania and Bulgaria, neighbouring countries in South-Eastern Europe that exhibit high heterogeneity in topography, climate, and land use. On a large scale, extensive mountainous areas with peaks up to 2500 m shape the face of both countries. In Romania, the Carpathian Mountains predominate [110], whereas in Bulgaria the landscape is structured by alternating bands of high and low terrain that stretch from east to west across the country [111]. The topography gives rise to various climatic zones ranging from alpine and subarctic to humid subtropical [110, 112]. The landscape is a mosaic of natural areas such as plains, open woodland, and extensive forests, as well as inhabited and in- and extensively used agricultural areas. This pronounced environmental heterogeneity provides an ideal study ground to identify subtle population structure and its drivers in a species believed to be quasi-panmictic.

### Field sampling

Over five consecutive years, from 2013 to 2017, we obtained 385 individuals from 25 locations across Romania and Bulgaria (Fig. 3(a)). Sampling sites (Additional file 1: Table S8) were at least 20 km apart to avoid overlapping foraging ranges [93, 113, 114]. Locations spanned a wide range of habitat conditions, encompassing both natural and semi-natural habitats, as well as extensive environmental gradients with respect to climate, vegetation and altitude. Sites were visited only once by a small team of 2–3 people for approximately 1.5 h each. Individuals regardless of sex were caught using insect nets and sacrificed in a jar with ethyl acetate [115]. Subsequently, specimens were transferred to individual tubes containing 96% ethanol and stored at −20 °C after returning to Tübingen University.

### Marker choice, DNA extraction, and genotyping

Even though single nucleotide polymorphisms are increasingly being used to address a plethora of evolutionary and ecological questions [116], microsatellites remain a powerful yet time and cost-efficient way of detecting population structure [117]. Indeed, the markers’ high mutation rates might render it particularly suited to infer the influence of recent ecological events on shaping spatial patterns of genetic diversity [118–120]. Additionally, genetic connectivity might be disrupted through reduced fitness of dispersers [9, 121]. Even though increasing genetic divergence is then caused by genetic drift, it is environmental differences that drive this differentiation in the first place. To this end, correlations between environmental variables and genetic divergence in neutral genetic markers, known as patterns of IBE/IBA, can be used to uncover hidden selective variation. Hence, microsatellites can be used to study the influence of the environment on shaping population structure in a widespread species such as *B. terrestris*. We acknowledge that this marker with its high allelic diversity within populations might depress Fst estimates [122]. Size homoplasy, where alleles are identical in state but not by descent might decrease Fst estimates even more but especially when dispersal among populations is low [123], which is unlikely to be the case in the highly vagile buff-tailed bumble bee. However, and regardless of the strength of the latter, both limitations render Fst values a conservative estimate of the true genetic divergence.

From each individual, we extracted DNA from one or two legs using the DNeasy Blood and Tissue Kit and QIAamp DNA Micro Kit (Qiagen, Hilden, Germany). We followed the manufacturer’s protocols except for adding 20 µl of 1 M dithiothreitol solution to each sample, which facilitates the extraction of DNA from chitinous materials [124]. Individuals of *B. terrestris,* specifically workers [125], can be difficult to distinguish morphologically in the field from another closely related bumble bee species, the white-tailed bumble bee (*Bombus lucorum)* [126]. We therefore confirmed species identity using a 1043 bp long fragment of the cytochrome c oxidase subunit I (CO1) gene [21].

We then amplified 12 previously developed [127] microsatellite loci in 3 multiplex reactions (PM1–PM3, Additional file 1: Table S1). PCR amplification was run in a total volume of 10 µl consisting of 5 µl PCR master mix (Qiagen), 2.1 µl HPLC H_2_O, 0.4 µl bovine serum albumin (10 mg/ml), 1 µl primer solution (100 µM, Applied Biosystems) and 1.5 µl sample DNA. Samples were initially denatured at 95 °C for 15 min, followed by 25 cycles of denaturation (94 °C, 30 s), annealing (PM1: 56 °C, PM2/3: 60 °C, both for 90 s) and extension (72 °C, 60 s). An additional 20 cycles were run using the following settings: denaturation (94 °C, 30 s), annealing (44 °C, 90 s) and extension (72 °C, 60 s). PCR products were visualised on an ABI3730XL capillary DNA sequencer (Applied Biosystems) using a GeneScan 500 LIZ size standard (Applied Biosystems) at Macrogen Europe (The Netherlands). Results were analysed using GeneMarker v.2.4.0 (SoftGenetics, State College, PA). Samples that had not amplified successfully or for which scoring had not yielded conclusive results were re-amplified and re-scored. Individuals that repeatedly failed to amplify or yielded inconclusive results for the second time were excluded. The presence of genotyping errors was assessed by re-amplifying and re-scoring 36 randomly selected samples, representing approximately 9.5% of all individuals. Scoring results were compared between the first and second run and the mean error rate for each locus was calculated in Microsoft Excel.

### Data analysis

#### Identification and removal of clones, sibling workers and drones

As the presence of clones and full siblings is known to distort estimates of population structure, we first identified clones using the ‘Multilocus Matches’ analysis in GenAlEx v.6.503 [42, 43]. Second, full siblings and additional clones were detected using the maximum likelihood approach implemented in Colony v.2.0.6.5 [44], which has been deemed the most reliable method for assigning sibship in bumble bees [34]. In brief, we assumed a polygamous mating system to allow Colony to infer the relationship among all individuals entered as offspring, as well as inbreeding [128], the presence of clones, and dioecious reproduction with haplodiploidy. Two runs, differing in their seed values, were conducted with medium length using the full likelihood method with medium precision. Dropout rate was set to 0.001 and locus-specific mean error rates ranged from 0 to 5.56%. Individuals were considered clones or full siblings when they were inferred in both runs with probabilities >0.8 and originated from the same population. For each inferred clone or full sibling pair, one randomly selected individual was retained, resulting in the so-called ‘complete data set’ (‘cpds’, Additional file 1: Table S8). As subtle genetic distances might not be inferred accurately for small populations [129], we excluded the populations ‘Billed’ and ‘Coastra’, resulting in a ‘mixed-ploidy data set’ (‘mpds’, Additional file 1: Table S8). Additionally, because haplodiploidy in *Bombus* (i.e. haploid males, diploid females) deflates measures of heterozygosity, we identified and excluded putative males in ‘mpds’ based on observed multilocus genotypes. More specifically, individuals were considered males if they were heterozygous for a maximum of two loci, resulting in a data set encompassing diploid individuals only (‘dpds’, Additional file 1: Table S8) from 21 populations throughout Romania and Bulgaria.

#### Population genetic analyses

After excluding putative clones, full siblings and haploid males, we tested for null alleles, stuttering and large allele dropout in Micro-Checker v.2.2.3 [45], applying 3000 randomisations and a Bonferroni correction while omitting missing data. Null alleles are those that do not reliably amplify in PCR, usually due to non-ideal conditions and/or mutations at primer-binding regions and result in heterozygotes appearing as homozygotes while homozygotes typically do not show alleles at the respective loci at all [130]. Furthermore, if the alleles in a heterozygote differ strongly in size, the shorter allele may be preferentially amplified during PCR, at the expense of the larger one. Hence, the signal of the larger allele might be too weak to be confidently detected during genotyping, resulting in the individual to be erroneously called as homozygous [131]. Hardy–Weinberg equilibrium (HWE) and genotypic linkage disequilibrium (LD) were assessed through Genepop on the Web v.4.2 using the Markov chain method with 10,000 dememorisations, 1000 batches and 10,000 iterations per batch. Subsequently, we used GenAlEx v.6.503 (‘dpds’) and SPAGeDi v.1.5 [132] (‘mpds’) to compute genetic diversity indices, including observed and unbiased expected heterozygosity. Rarefied allelic richness [133] was obtained in hp-rare v.1.1 [134] (‘dpds’) and SPAGeDi (‘mpds’). We then computed population pairwise and global genetic distances (Fst) and corresponding *P* values for ‘dpds’ using GenAlEx with 9999 permutations. Negative Fst values were converted to zero and *P* values were adjusted for multiple testing through false discovery rate (FDR) correction [47] using the ‘p.adjust’ function in R v.3.6.0 [135]. For ‘mpds’ both the Fst matrix and global Fst value were computed with the ‘calcPopDiff’ function based on allele frequencies calculated with the ‘simpleFreq’ function in the polysat v.1.7.4 R package [48]. Additionally, for both global and pairwise values, we computed the 95% confidence intervals of 10,000 bootstrap replicates. Fst values were considered significant if they surpassed the upper 95% confidence interval. To explore whether potential differences in Fst estimates between ‘dpds’ and ‘mpds’ are simply an artefact of the different computational approaches used, we also calculated pairwise and global Fst estimates for ‘dpds’ in polysat. Significance testing was performed as described above.

Following the computation of estimates of Fst, we assessed genetic structure in ‘cpds’ through Bayesian clustering in Structure v.2.3.4 [1] using the ‘Admixture’ and ‘Correlated Allele Frequency’ models with population IDs as priors [51]. We set the number of clusters (*K*) from 2 to 25 (number of populations sampled) and computed 5 iterations with a burnin period of 100,000 and 1,000,000 Markov chain Monte Carlo (MCMC) repetitions. Relative admixture between populations was estimated by Structure (INFERALPHA = 1). Because previous studies suggested that buff-tailed bumble bees are quasi-panmictic, and they are highly vagile, we hypothesised that admixture is common, and the corresponding model in Structure was biologically most meaningful. Yet, we also explored the performance of Structure’s ‘No admixture’ model by running two iterations with the aforementioned burnin period and MCMC repetitions. Additionally, as uneven sampling sizes among populations might result in an incorrect number of clusters [136], two further Structure analyses were run after excluding populations with fewer than 10 individuals (‘pop10’, Additional file 1: Table S8). After setting the maximum *K* to 23, the first run was computed with the settings specified above while for the second run we additionally set STARTATPOPINFO to 1 to initialise each individual at its own population [137]. Structure output was visualised with the pophelper v.2.3.1 R package [138].

As complex patterns of population subdivision might not be detected reliably using only one clustering approach, we complemented Structure runs with discriminant analysis of principal components (DAPC, 40) implemented in the adegenet v.2.1.5 package [139] in R v.4.1.1. In fact, by maximising between-group variability, DAPC might be well-suited to detect suble and complex patterns of population subdivision, such as hierarchical clustering or clines [40]. We performed two DAPC runs. Briefly, the first one was computed using populations as a priori groups. For the second analysis, we inferred clusters de novo using the ‘find.clusters’ function with 1,000,000 iterations and 700 random starting points, and selected the number of clusters that minimised the BIC value. To avoid overfitting, we determined the optimal number of PCs to retain in both analyses using the ‘optim.a.score’ function with 1000 simulations, respectively. The results were visualised in scatter plots.

#### Mechanisms of divergence

As the likelihood of successful dispersal between populations decreases with geographic distance, gene flow starts to become limited and populations diverge through genetic drift. Under this scenario, a positive relationship between geographic and genetic distance is anticipated, a pattern termed isolation by distance (IBD, 7). Moreover, heterogeneous conditions of the habitat through which dispersal takes place impose varying levels of resistance to dispersing individuals. They may therefore not follow a straight line, but instead the path of least resistance. Least-cost path [140] and isolation by resistance (IBR, 8) analyses aim to quantify this heterogeneity in habitat permeability and its effect on population structure. Both IBD and IBR describe processes resulting in neutral population divergence, and are jointly coined isolation by dispersal limitation [141]. In addition, species experience heterogeneous environmental conditions that may exert strong selection pressures on populations, potentially leading to local adaptation and hence population divergence [142, 143]. Interestingly, the prolonged reduced fitness of dispersers [9] that are maladapted to newly encountered conditions might result in the disruption of genetic connectivity. As a consequence, populations may differentiate by means of genetic drift in neutral markers, a pattern termed isolation by environment/ecology (IBE, [10, 11]) or, alternatively, isolation by adaptation (IBA, [12]).

We considered all three potential mechanisms of population divergence in our analyses. In addition to straight-line geographic distance, we also included a measure of habitat permeability using resistance distances based on a previously published species distribution model [21], as well as a set of environmental variables.

#### Environmental variables and permeability of the habitat matrix to dispersal

We characterised environmental conditions across Romania and Bulgaria using a set of 16 environmental variables compiled for previous species distribution and landscape genetic studies [21, 39] (Additional file 1: Table S9). This set included variables related to climate, vegetation and topography at 30 arcseconds resolution, which roughly converts to a 1 km resolution at the equator. Briefly, we initially considered 19 climate variables from WorldClim v.2 [144], including temperature and precipitation variables based on a 30-year climatology from 1970 to 2000 [145]. Elevation data originated from the Shuttle Radar Topography Mission (SRTM, [146]), and were also used to compute aspect and slope. We included spatial and temporal vegetation patterns derived from satellite data: percent tree cover and Leaf Area Index (the one-sided green leaf area per unit ground area), both obtained from the Global Land Cover Facility database [147]. Information on vertical forest structure, i.e. canopy height, was derived from space borne LiDAR from 2011 [148]. Finally, to incorporate information about surface moisture, we included annual mean, minimum, maximum, and seasonality, computed from raw QuikSCAT data [39]. To do so, we used daily raw backscatter measurements downloaded from the BYU Scatterometer Climate Record Pathfinder database [149] over the period the instrument was online (2000–2008). This initial data set was reduced by excluding highly correlated variables. We did so using their variance inflation factor (VIF) and excluded those with a score ≥ 10 in a stepwise fashion in the usdm R package v.1.1–18 [150] in R v.3.6.1. To facilitate the discussion of our findings, we also quantified environmental similarities among population locations by means of Pearson correlation coefficients (Additional file 1: Table S7), calculated using the ‘cor’ function in R v.3.6.2. More information on the environmental data and processing can be found elsewhere [21, 39].

To obtain a measure of habitat permeability, we further calculated population pairwise resistance distances based on the species distribution model (SDM) of *B. terrestris* [21]. Computations were carried out in Circuitscape v.4.0.5 [151] with the SDM surface being treated as a conductance map, a cell size of 0.0083333° (i.e. 30 arcseconds) and a cell connection scheme of eight neighbours. Although species distribution models are not directly informative about genetic connectivity (i.e. functional connectivity, [152]), they represent the spatial configuration of putatively suitable habitat (i.e. structural connectivity, [153]). One caveat of this approach is that habitat unsuitable for breeding or foraging may nevertheless be easily crossed by vagile species. Yet, it may be reasonable to suspect that more suitable habitat can be crossed more easily than less suitable habitat, and thus that SDMs provide a useful estimate of relative habitat permeability. We therefore considered it meaningful to use SDM-derived population pairwise resistance distances (Additional file 1: Table S10) as additional predictors in our analyses.

### Landscape genetic analyses

We established associations between genetic data and environmental variables using generalized dissimilarity modelling implemented in the gdm R package v.1.3.11 [154]. This extension of the classical matrix regression allows to fit non-linear relationships between predictors and response variables. Additionally, the contribution of predictors can be analysed simultaneously [41]. In a landscape genetics framework this means that the effects of geographic distance and the environment on genetic divergence can be analysed at the same time, and estimates of variable importance are provided through permutation [155–157]. Predictors inferred as informative in explaining genetic turnover yield I-splines that provide two pieces of information. The maximum height of the curve indicates the amount of biological change along the gradient, while the spline’s shape informs about the rate of genetic turnover [156]. In this study, we used this framework to infer the relationship between pairwise genetic distances (Fst, scaled between zero and one) and environmental variables, geographic distance and resistance distance. In total, we computed five different models, that included (1) environmental variables, geographic and resistance distances (full model), (2) environmental variables only, (3) straight-line geographic distances only, (4) resistance distances only, and (5) 1000 models, each using 16 random environmental variables to evaluate the performance of the full model. We considered the full model significant if its variation explained surpassed the 95% confidence interval of the random models. Subsequently, following the approach by Fitzpatrick and Keller [156], we used the inferred relationships between predictor and response variables at sampling sites to predict genetic turnover across Romania and Bulgaria. First, for each retained environmental variable, we extracted its value across the study area at 30 arcseconds resolution. Using the ‘gdm.transform’ function from the gdm package, we then transformed environmental variables into ‘genetic’ importance values. We selected the three most influential and uncorrelated factors through principal component analyses (PCA) using the ‘princomp’ function with calculations performed on the covariance matrix. We purposely centred, but not scale-transformed the PCA to preserve differences in the magnitude of the genetic importance among environmental variables. With the ‘rasterize’ function in QGIS v.3.16.2 [158] we converted the obtained point values to rasters, merged them with the ‘r.composite’ function to a composite and assigned the corresponding PC scores to a RGB palette. The result (Fig. 3(b)) visualises differences in genetic composition, where increasingly dissimilar colours represent higher predicted genetic turnover.

## Supplementary Information

**Additional file 1: Table S1. **Microsatellite loci used in this work, with original locus names [127], the primer mixes they were assigned to, repeat units (motifs), size ranges, annealing temperatures (T_m_), forward and reverse primer sequences (5’–3’) as well as information on null alleles and stuttering. **Table S2.** Basic population genetic statistics for each sampling location encompassing diploid (‘dpds’) and both haploid and diploid individuals (‘mpds’). N: number of individuals; N_A_: number of alleles; N_E_: number of effective alleles; A_R_: allelic richness, rarefied for seven individuals (k); uH_E_: unbiased expected heterozygosity; H_O_: observed heterozygosity. Observed heterozygosity values between ‘dpds’ and ‘mpds’ are largely congruent, as SPAGeDi, even though capable of working with mixed-ploidy data sets, does not include haploid individuals in calculating this metric. **Table S3.** Below the diagonal: Multilocus pairwise Fst values calculated with GenAlEx and based on a data set containing diploid individuals only. Above the diagonal: Corresponding FDR-corrected *P* values. Marginally significant Fst values and *P *values are indicated in bold. See Table S8 for population names. **Table S4.** Above the diagonal: Multilocus pairwise Fst values computed with polysat and based on a data set containing both haploid and diploid individuals. Below the diagonal: Corresponding bootstrap-derived upper 95% confidence intervals. See Table S8 for population names. **Table S5.** Above the diagonal: Multilocus pairwise Fst values calculated with polysat and based on a data set containing diploid individuals only. Below the diagonal: Corresponding bootstrap-derived upper 95% confidence intervals. See Table S8 for population names. **Table S6**. Discriminant analysis of principal components (DAPC) result showing the assignment of individuals to clusters identified de novo. **Table S7.** Pearson correlation coefficients of environmental variables at population locations harbouring both haploid and diploid individuals. See Table S9 for the abbreviations used. **Table S8.** Populations sampled, including their abbreviations, locations, and the number of individuals. ‘cpds’: full data set after exclusion of full siblings and clones; ‘pop10’: ‘cpds’ after excluding populations with fewer than 10 individuals; ‘mpds’: mixed-ploidy data set encompassing both haploid males and diploid females; ‘dpds’: data set containing diploid individuals only. **Table S9.** Environmental variables used. Those in bold were retained after stepwise elimination with a variance inflation factor ≥ 10. **Table S10.** Resistance matrix derived from Circuitscape, where higher values indicate higher predicted resistance to inter-population dispersal. See Table S8 for population names.

**Additional file 2:** Structure output based on the ‘complete data set’ (‘cpds’) for selected values of *K*; result of the a-score optimisation for the DAPC runs performed as well as the DAPC result obtained using populations as a priori groups; a curve depicting model performance for different numbers of clusters inferred de novo; GDM splines produced from data sets encompassing haploid and diploid individuals (mixed-ploidy data set’, ‘mpds’) and diploid individuals only (‘diploid data set’, ‘dpds’).

## Data Availability

The datasets generated and/or analysed during the current study are available in the Dryad repository https://doi.org/10.5061/dryad.gb5mkkwq8. The R scripts used for data processing are available from the corresponding author upon reasonable request.

## Abbreviations

A_R_: Allelic richness
CI: Confidence interval
CO1: Cytochrome c oxidase subunit I
cpds: Complete data set; a data set containing both haploid and diploid individuals before any populations were excluded due to low sample sizes
DAPC: Discriminant analysis of principal components
dpds: Diploid data set; a data set containing diploid individuals only
H_O_: Observed heterozygosity
*K*: Number of Structure clusters
k: Number of individuals used for calculating rarefied allelic richness
mpds: Mixed-ploidy data set; a data set containing both haploid and diploid individuals
msX: Microsatellite loci X
N: Number of individuals
N_A_: Number of alleles
N_E_: Number of effective alleles
PMX: Primer mix X
pop10: Complete data set after excluding populations with fewer than 10 individuals
T_m_: Annealing temperature
uH_E_: Unbiased expected heterozygosity
