# Supplementary material for: Environmental differences explain subtle yet detectable genetic structure in a widespread pollinator
Source: BMC Ecol Evol. 2022 Feb 1;22:8. doi: 10.1186/s12862-022-01963-5 (PMC8808969; doi:10.1186/s12862-022-01963-5)
Supplement: Supplementary file 2 — Additional file 2: Structure output based on the ‘complete data set’ (‘cpds’) for selected values of K; result of the a-score optimisation for the DAPC runs performed as well as the DAPC result obtained using populations as a priori groups; a curve depicting model performance for different numbers of clusters inferred de novo; GDM splines produced from data sets encompassing haploid and diploid individuals (mixed-ploidy data set’, ‘mpds’) and diploid individuals only (‘diploid data set’, ‘dpds’). [file 12862_2022_1963_MOESM2_ESM.docx]

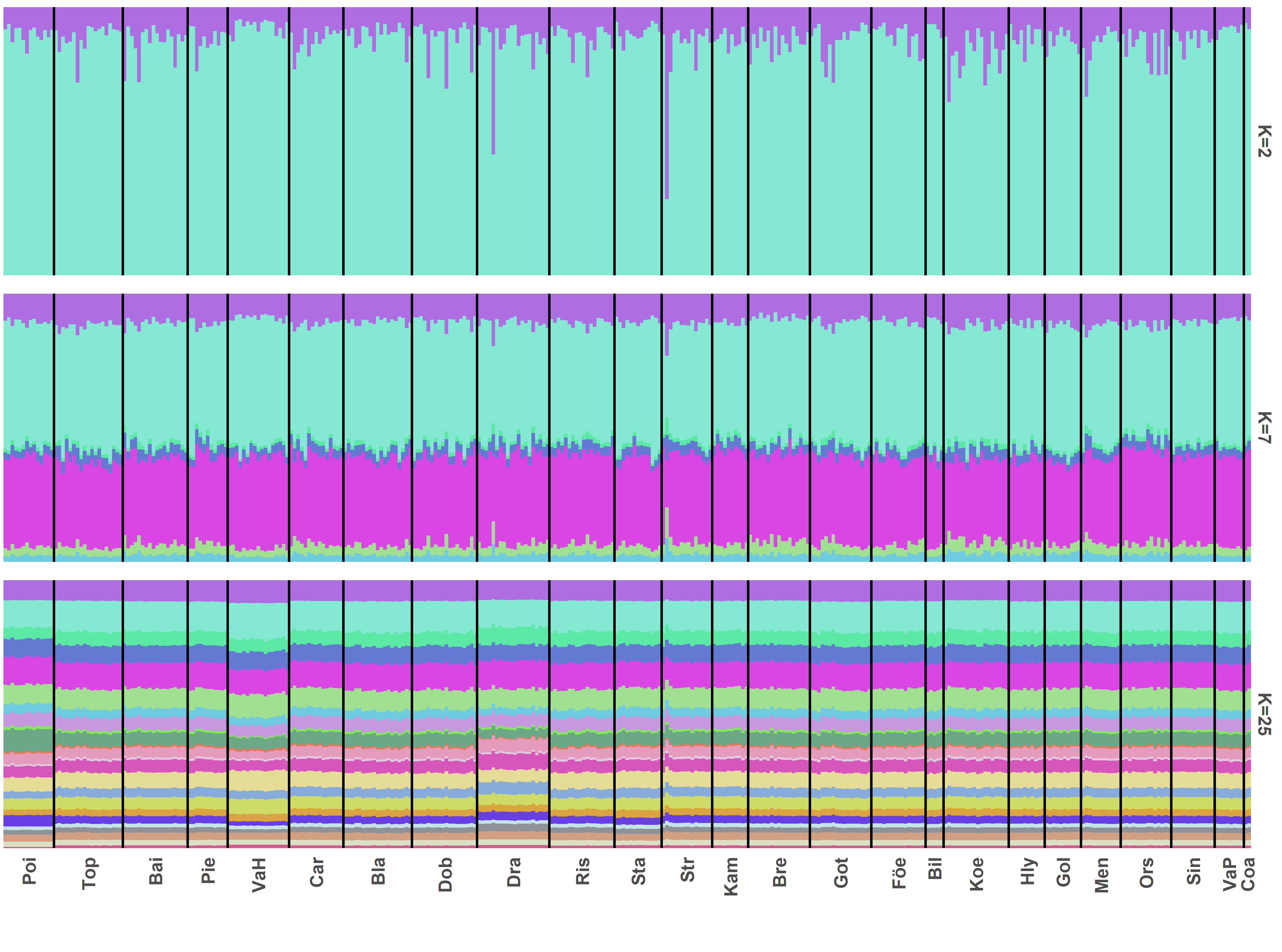


**Figure S1:** Structure results based on ‘cpds’ and derived using the ‘Admixture model’ for *K* = 2, 7, and 25. Each vertical bar represents one individual, with the proportional height of each colour indicating the posterior probability of membership to the corresponding cluster. As the five independent runs for each *K* yielded congruent results, the graphical representation of only one of the five independent runs is presented. See Additional file 1: Table S8 for population names.


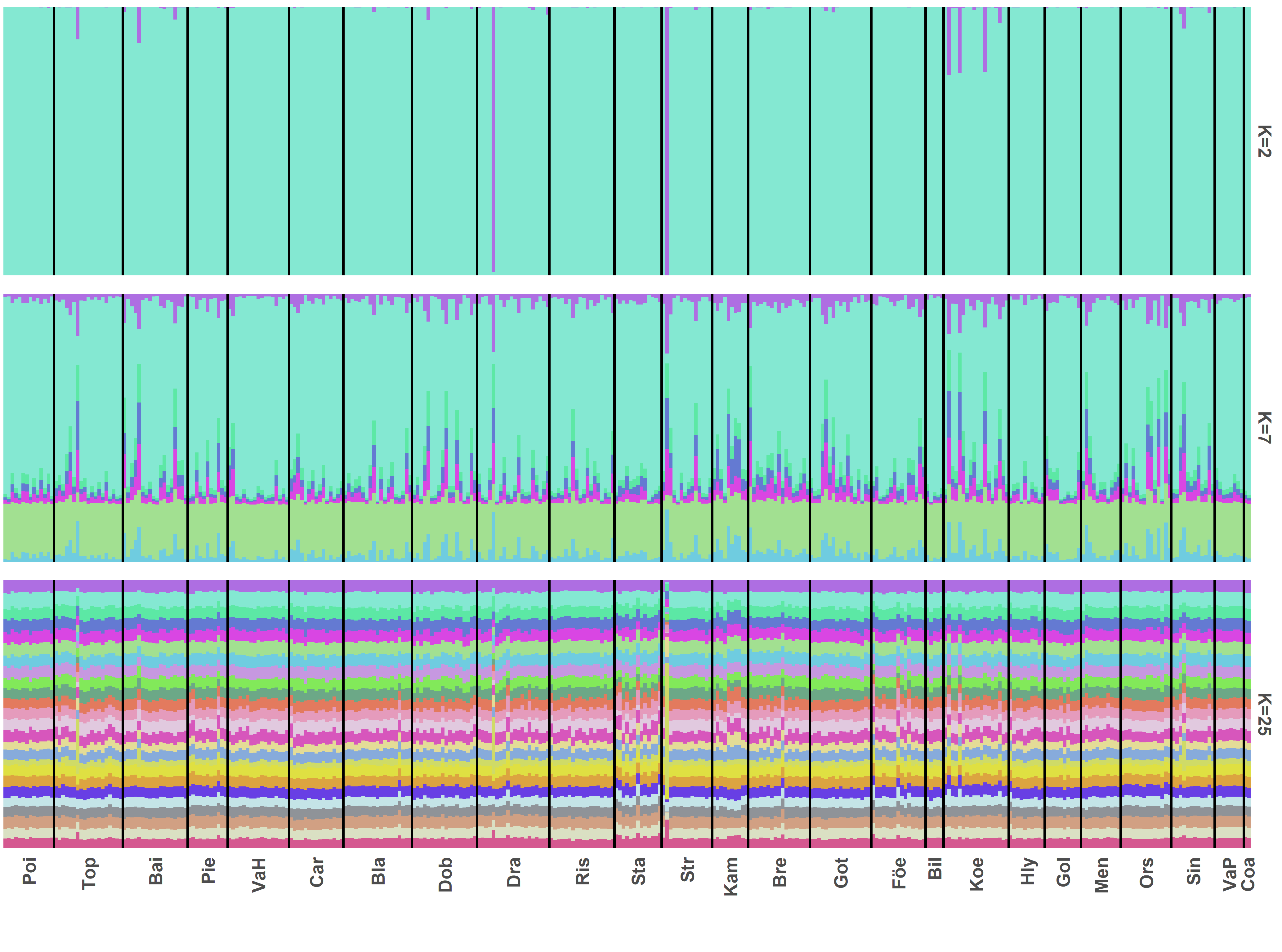
Figure S2: Structure results based on ‘cpds’ and derived using the ‘No admixture model’ for *K* = 2, 7, and 25. Each vertical bar represents one individual, with the proportional height of each colour indicating the posterior probability of membership to the corresponding cluster. As the two independent runs for each *K* yielded congruent results, the graphical representation of only one of the two independent runs is presented. See Additional file 1: Table S8 for population names.


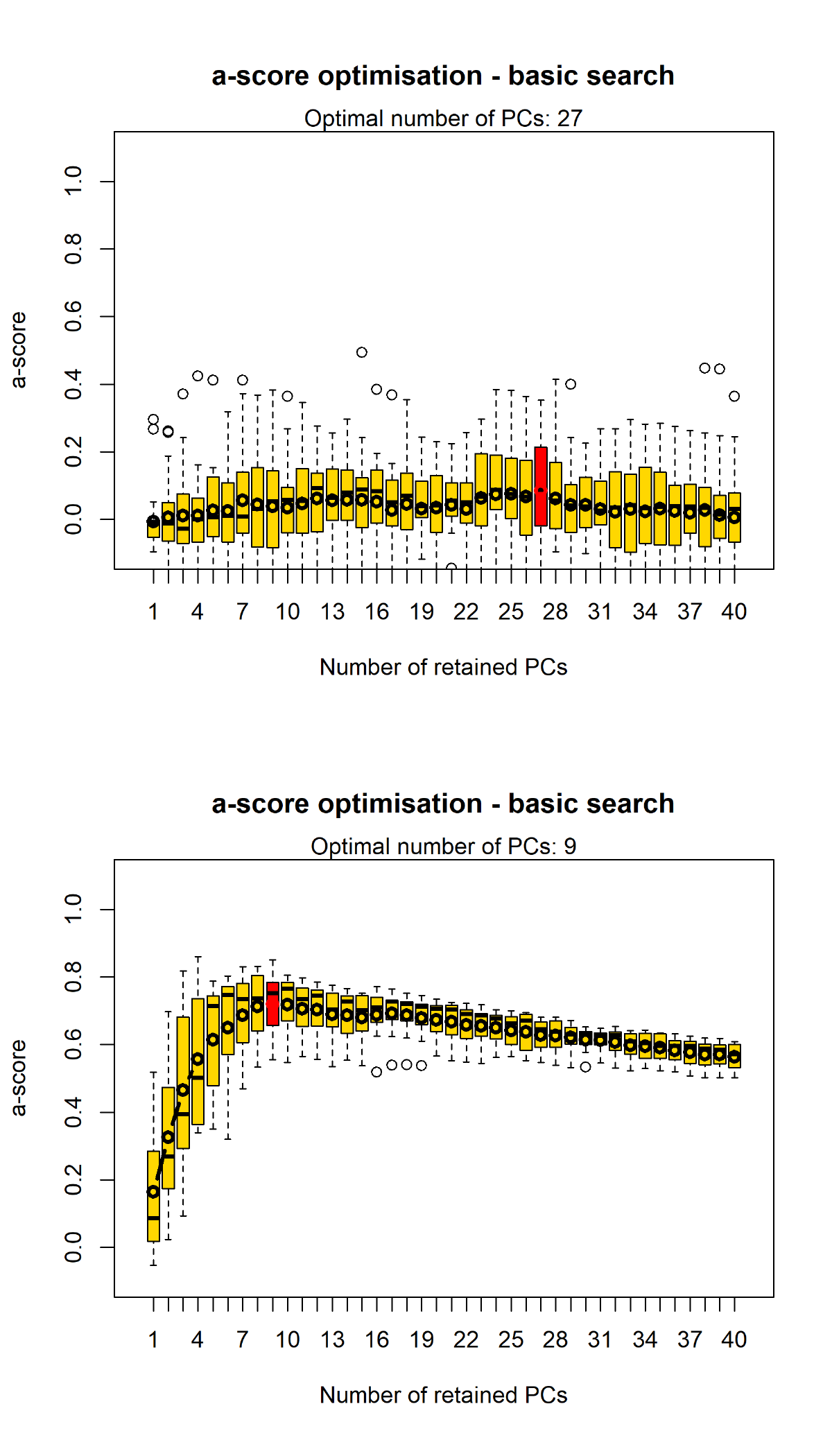


**Figure S3:** Results of a-score optimisation using the ‘optim.a.score’ function for discriminant analysis of principal components (DAPC) runs using populations as *a priori* groups (top) and clusters inferred *de novo* using the “find.clusters” function (bottom).


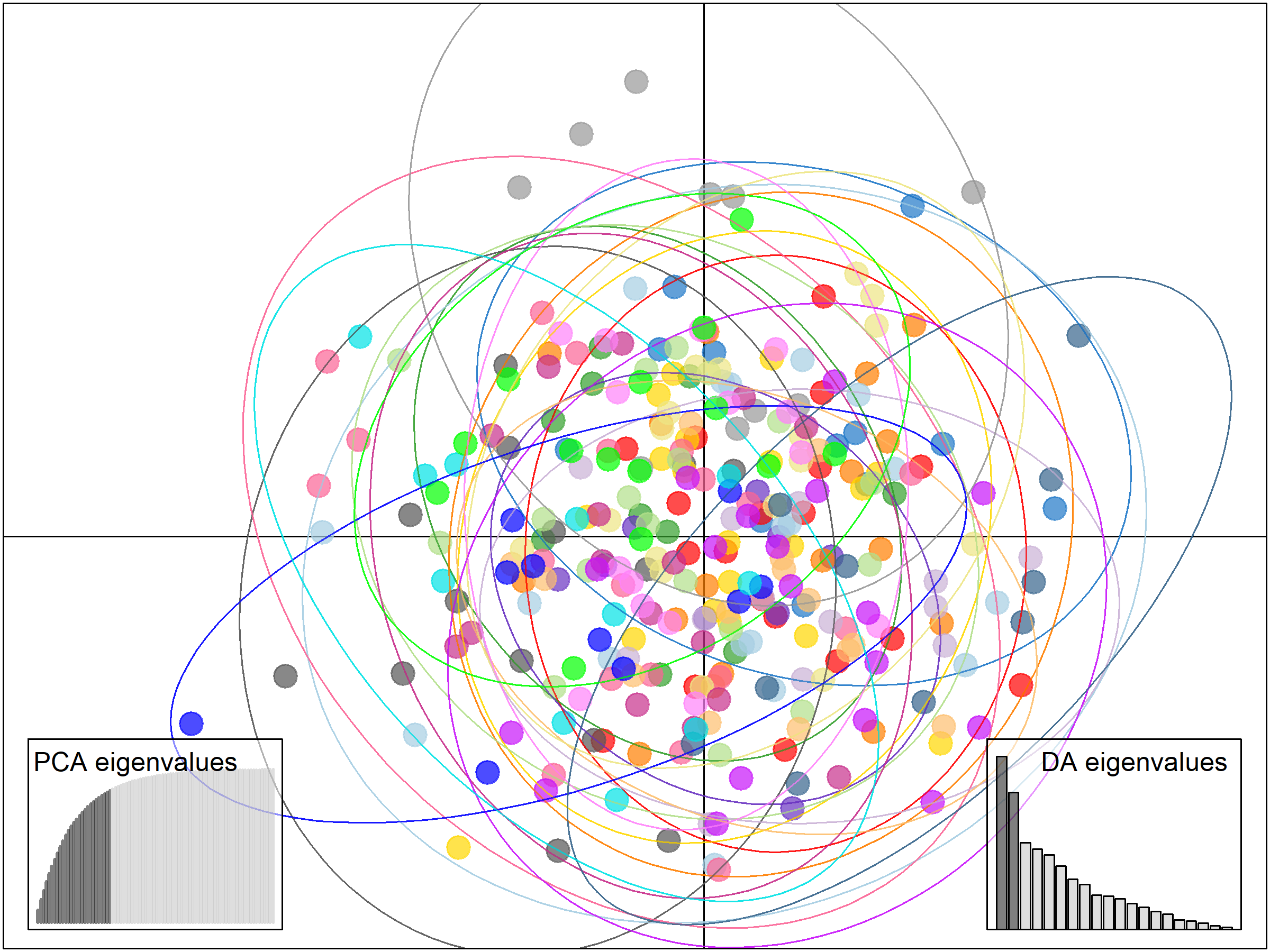


**Figure S4:** Scatter plot of the discriminant analysis of principal components (DAPC) using populations as *a priori* groups. 27 principal components (PCs) were retained to avoid overfitting; mean a-score was 0.04. Ellipses indicate the 95% interval of assignment. Insets depict the principal component analysis (PCA) and discriminant analysis (DA) eigenvalues. Highlighted bars in insets show the number of PCs retained and the discriminant functions visualised, respectively.


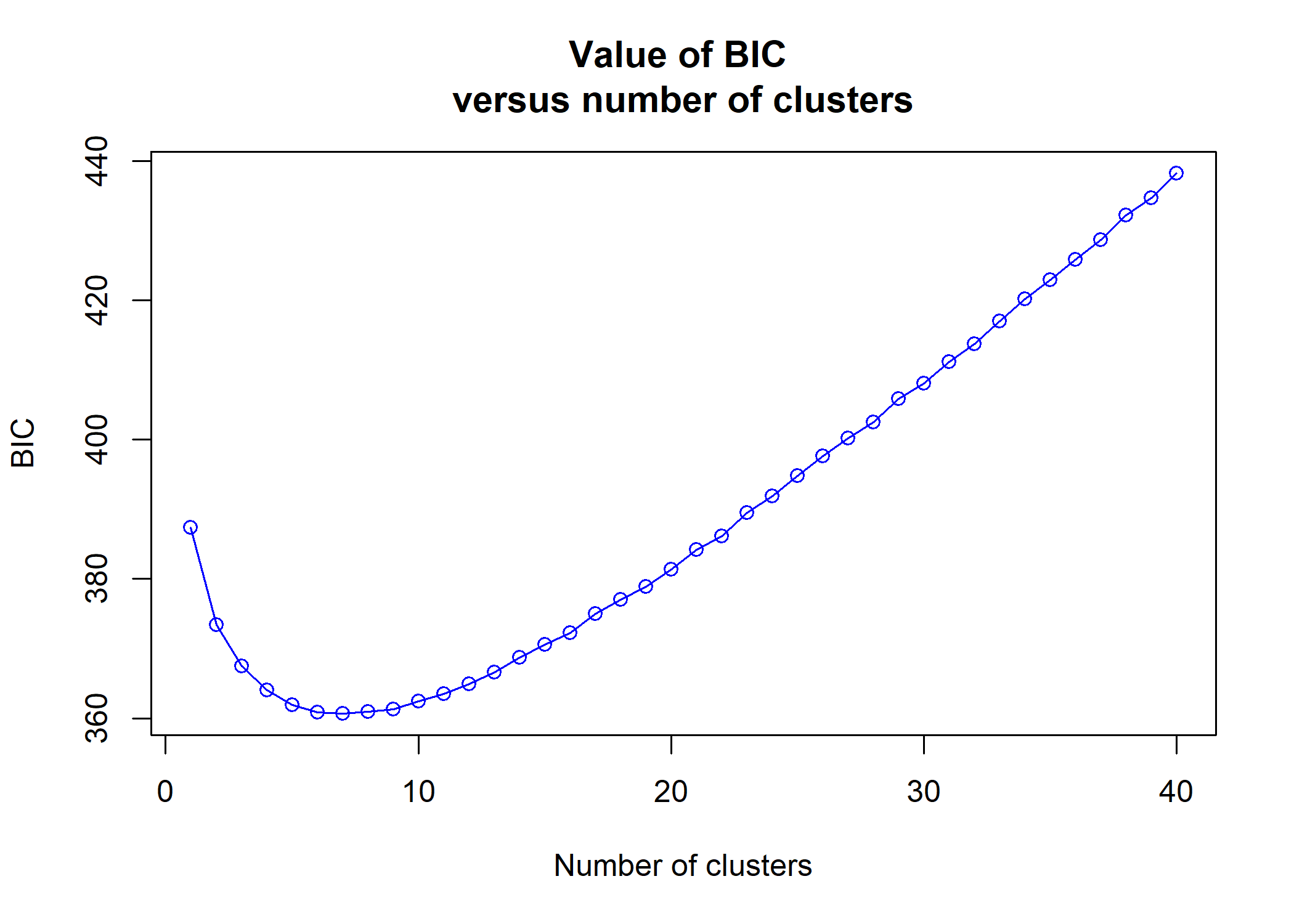


**Figure S5:** BIC values for different numbers of clusters inferred *de novo* using the ‘find.clusters’ function.


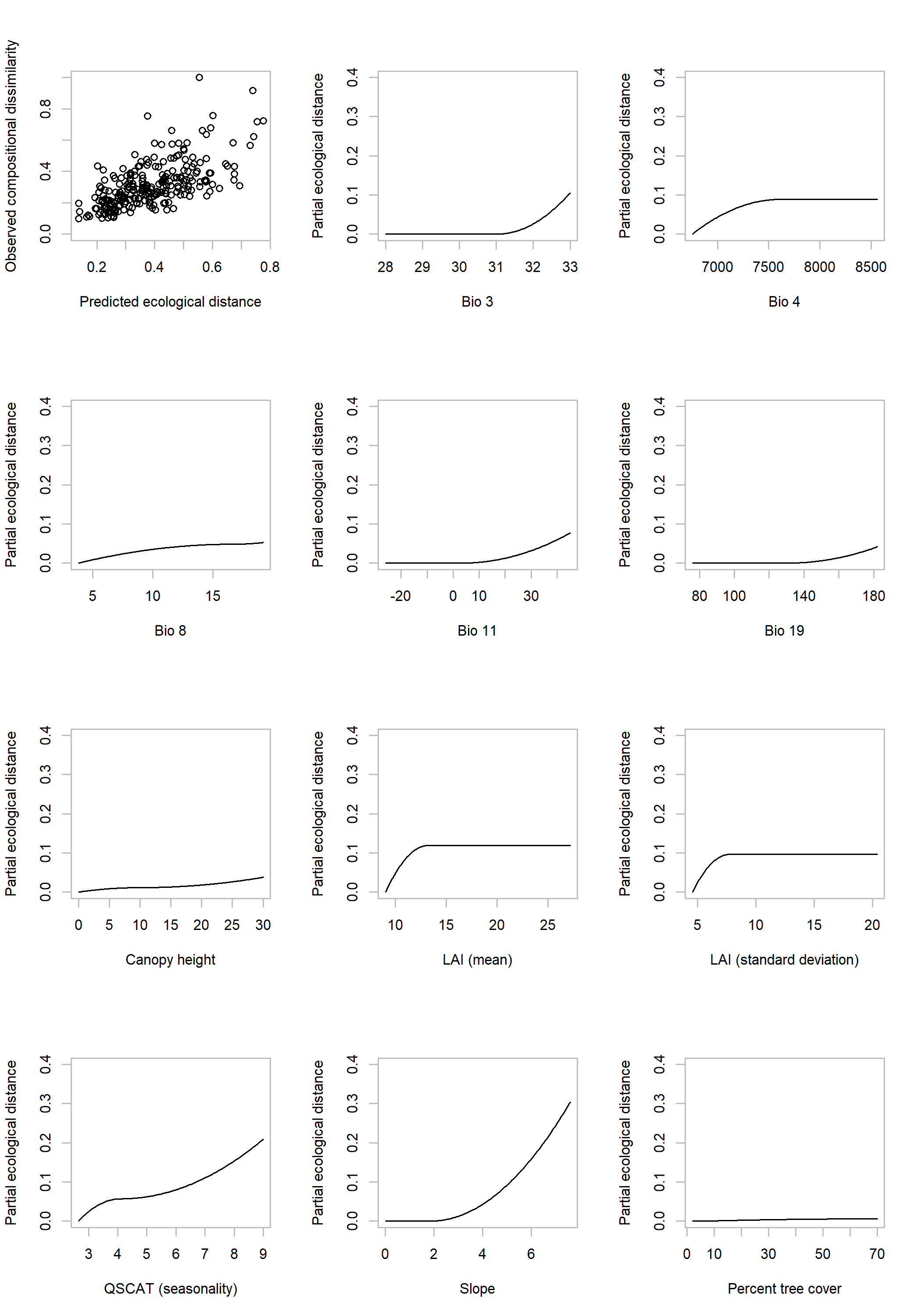
 Figure S6: Splines derived from the full generalized dissimilarity model (GDM) after excluding variables with a variance inflation factor ≥ 10. Underlying Fst values were computed from the data set including di- and haploid individuals (‘mpds’). Top left plot: Plotted relationship between predicted ecological and observed compositional dissimilarity. Remaining plots: Visualisation of how the selected variable contributed to the observed genetic turnover. The maximum height reached by each curve indicates the amount of variation explained; its slope is indicative for the rate of change in genetic composition.


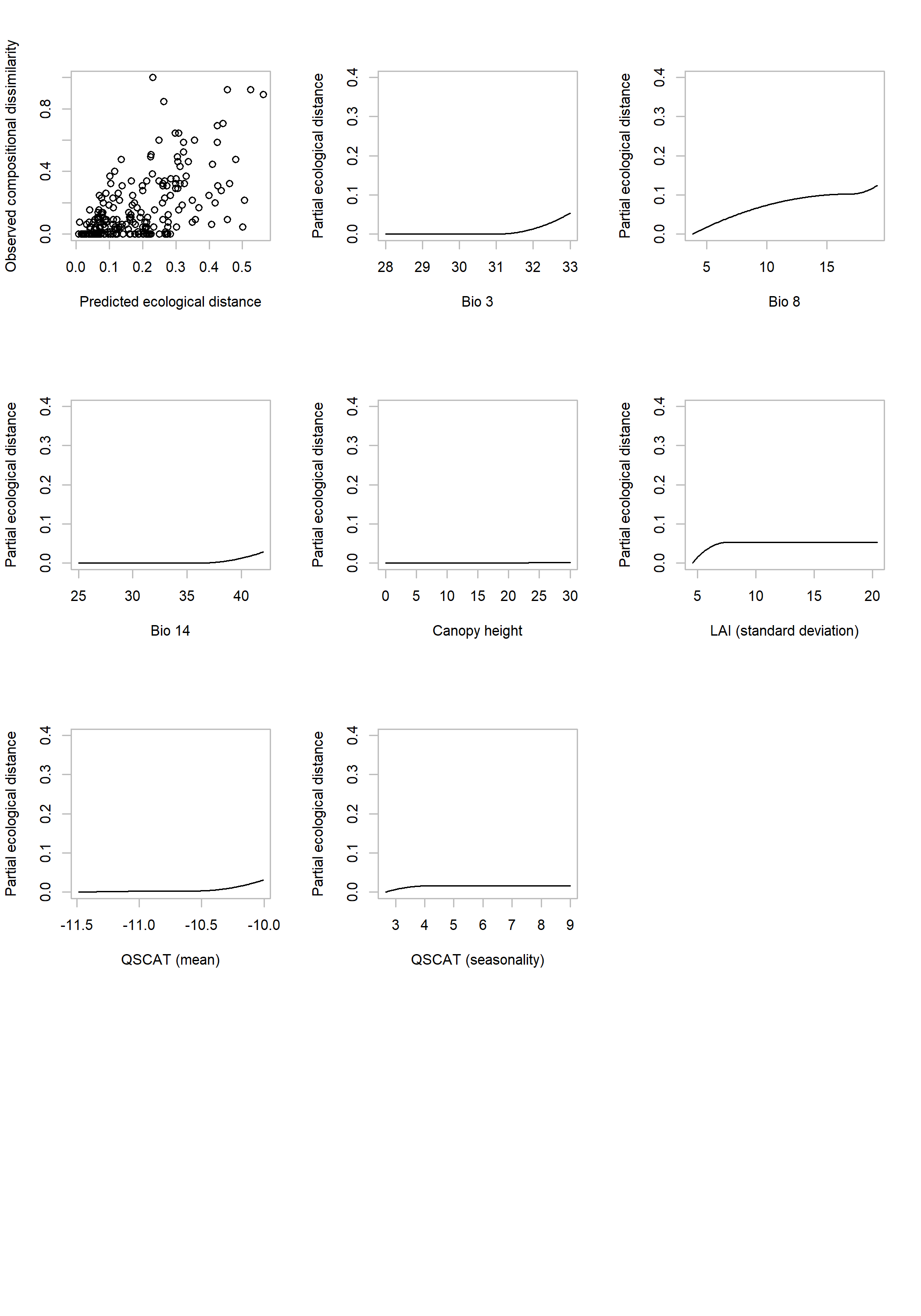


Figure S7: Splines derived from the full generalized dissimilarity model (GDM) after excluding variables with a variance inflation factor ≥ 10. Underlying Fst values were computed from the data set including diploid individuals only (‘dpds’). Top left plot: Plotted relationship between predicted ecological and observed compositional dissimilarity. Remaining plots: Visualisation of how the selected variable contributed to the observed genetic turnover. The maximum height reached by each curve indicates the amount of variation explained; its slope is indicative for the rate of change in genetic composition.
